# Prevalence of Headaches and Their Subtypes Among Medical Students: A Cross-Sectional Survey at Bahcesehir University, Turkey

**DOI:** 10.7759/cureus.73831

**Published:** 2024-11-16

**Authors:** Alara Abu Saadeh, Houssam Eddine Youcefi, Buse Cagla Ari, Muhammad Hamza Dawood, Dana Abou Shaar, Ali Kimiaei, Seyedehtina Safaei, Ahmet Midi

**Affiliations:** 1 Neurology, Bahçeşehir University, Istanbul, TUR; 2 Medicine, United Medical and Dental College (UMDC), Karachi, PAK; 3 Pathology, Bahçeşehir University, Istanbul, TUR

**Keywords:** headache, headache features, medical school students, prevalence rate, turkey

## Abstract

This study aimed to assess headache prevalence and the impact of lifestyle choices among medical students at Bahçeşehir University, Istanbul, Turkey. A cross-sectional survey of 180 participants was conducted, revealing that 78.80% reported headache occurrences. Tension-type headaches were the most common, with a prevalence rate of 59.50%. Lifestyle factors, such as irregular sleep schedules, dissatisfaction with academic performance, lack of exercise, and the use of electronic devices for studying, were correlated with headache occurrence. These findings underscore the need for tailored interventions to enhance sleep patterns, manage stress, promote balanced lifestyle habits, and encourage physical activity among medical students.

## Introduction

Headaches are a prevalent and often debilitating condition experienced by millions of individuals worldwide. They represent a significant public health concern due to their frequency, impact on quality of life, and economic burden. Numerous population-based studies have explored headache prevalence broadly [1-6], yet there is a notable gap in understanding its frequency and characteristics among specific groups, especially medical students in Turkey. We aim to examine the prevalence of headache disorders among medical students at Bahçeşehir University, Istanbul, Turkey, and investigate the potential impact of their lifestyle choices on headache susceptibility. Medical students represent a unique demographic encountering rigorous academic schedules, inadequate sleep, and heightened levels of stress. By conducting a comprehensive survey among medical students, our objective is to gather significant data on the prevalence of headache disorders within this specific student population at Bahcesehir University. Furthermore, we aim to study the potential correlation between lifestyle factors, including sleep patterns, stress levels, dietary habits, and physical activity, and the frequency and severity of headaches. The outcomes of this investigation hold significant implications for both medical students and the wider medical community. Gaining insight into the frequency of headaches and their correlated lifestyle factors among medical students could highlight potential areas of concern and guide interventions that aim to enhance their well-being and academic performance.

## Materials and methods

This cross-sectional analysis study was conducted at Bahçeşehir University in Istanbul, Turkey, from July 2023 to January 2024. Participation in the study was entirely voluntary, and confidentiality was assured. To minimize selection bias, the survey was distributed electronically to all students in the first to sixth years of our six-year curriculum via the university’s official email system, responses were collected anonymously, and standardized questions were used; additionally, all participants were required to provide consent before completing the survey. The sample size of 300 participants was determined based on an estimated response rate for the survey, ensuring a sufficient number of responses for meaningful statistical analysis.

We deployed a self-administered questionnaire divided into three sections: demographic data, headache characteristics, and alleviating factors. The demographic section was designed to examine the lifestyle factors common among medical students suffering from headaches and to compare the prevalence and etiology of headaches between male and female cohorts. The headache characteristics section aimed to gather detailed information about the type, frequency, duration, and intensity of headaches experienced by students, as well as potential triggers and their impact on daily activities. The alleviating factors section explored the strategies students use to manage their headaches, including both pharmacological (e.g., medications) and non-pharmacological approaches (e.g., rest, hydration). For content validity, the questionnaire was developed by two of the authors and further verified by a neurology specialist. The Bahçeşehir University Ethics Committee approved the study protocol (document no:2023-12/01), ensuring adherence to ethical standards. The survey’s design, although concise, was sufficiently detailed to capture the necessary information for analysis. For the diagnosis of headache types, the criteria from the International Classification of Headache Disorders (ICHD) were applied. Exclusion criteria were applied to maintain the integrity of the data, and students were excluded from the final analysis if they did not complete the survey; had a medical history of head trauma, tumors, and systemic or chronic diseases; or did not provide consent. These exclusions ensured that the study results would be representative of the student population, free from confounding medical conditions.

Responses were recorded in an Excel sheet (Microsoft® Corp., Redmond, WA) and then coded and imported into Statistical Product and Service Solutions (SPSS, version 26; IBM SPSS Statistics for Windows, Armonk, NY) for analysis. Frequencies and percentages were reported for categorical variables. Fisher's exact test was applied to determine the prevalence of headaches, while the chi-square test was used to assess the relationship between headache prevalence and demographic characteristics, as well as the association between headache types and demographic, academic, and lifestyle factors. A p-value of less than 0.05 was considered statistically significant.

## Results

Demographic, academic, and lifestyle characteristics of participants

Out of the 300 students initially enrolled in the study, 180 completed the questionnaire. Of these, 20 participants were excluded from the analysis due to a confirmed diagnosis of secondary causes of headaches. The mean age of the participants was 21 years, with an age range of 17-27 years. Among the 160 participants included in the analysis, there was an equal distribution of sex and academic year. Detailed academic and lifestyle characteristics are presented in Table 1.

**Table 1:** Demographic and lifestyle characteristics of participants (n=160)

Variables	n (%)
Sex	
Male	78 (48.8%)
Female	82 (51.2%)
Academic Year	
1st year	12 (7.5%)
2nd year	55 (34.4%)
3rd year	20 (12.5%)
4th year	21 (13.1%)
5th year	15 (9.4%)
6th year	37 (23.1%)
Study Hours/Week	
<7 hours	20 (12.5%)
14-28 hours	74 (46.3%)
35-42 hours	44 (27.5%)
> 42 hours	22 (13.8%)
Late Night Study Frequency	
Never	33 (20.6%)
Sometimes	98 (61.3%)
Always	29 (18.1%)
Academic Performance Satisfaction	
Yes	158 (98.8%)
No	2 (1.3%)
Usage of Internet Devices for Study	
Yes	158 (98.8%)
No	2 (1.3%)
Eating and Drinking Habits	
I follow a vegan diet	4 (2.5%)
I follow a keto diet	3 (1.9%)
I usually eat home cooked meals	83 (51.9%)
I eat fast food >4 times per week	42 (26.3%)
I don't follow any particular diet	94 (58.8%)
I drink >2 cups of coffee per day	54 (33.8%)
I drink >2L of water per day	74 (46.3%)
Sleeping Habits	
I have problems falling asleep	44 (27.5%)
I have problems staying asleep	19 (11.9%)
I sleep less than 6 hours per day	38 (23.8%)
I sleep 6-8 hours per day	98 (61.3%)
I sleep more than 8 hours	30 (18.8%)
Exercise Frequency	
1-3 hours per week	57 (45.2%)
4-6 hours per week	27 (21.4%)
6-9 hours per week	13 (10.3%)
>10 hours per week	5 (4.0%)
Never	24 (19.0%)

Prevalence of headaches and their subtypes

**Figure 1:**
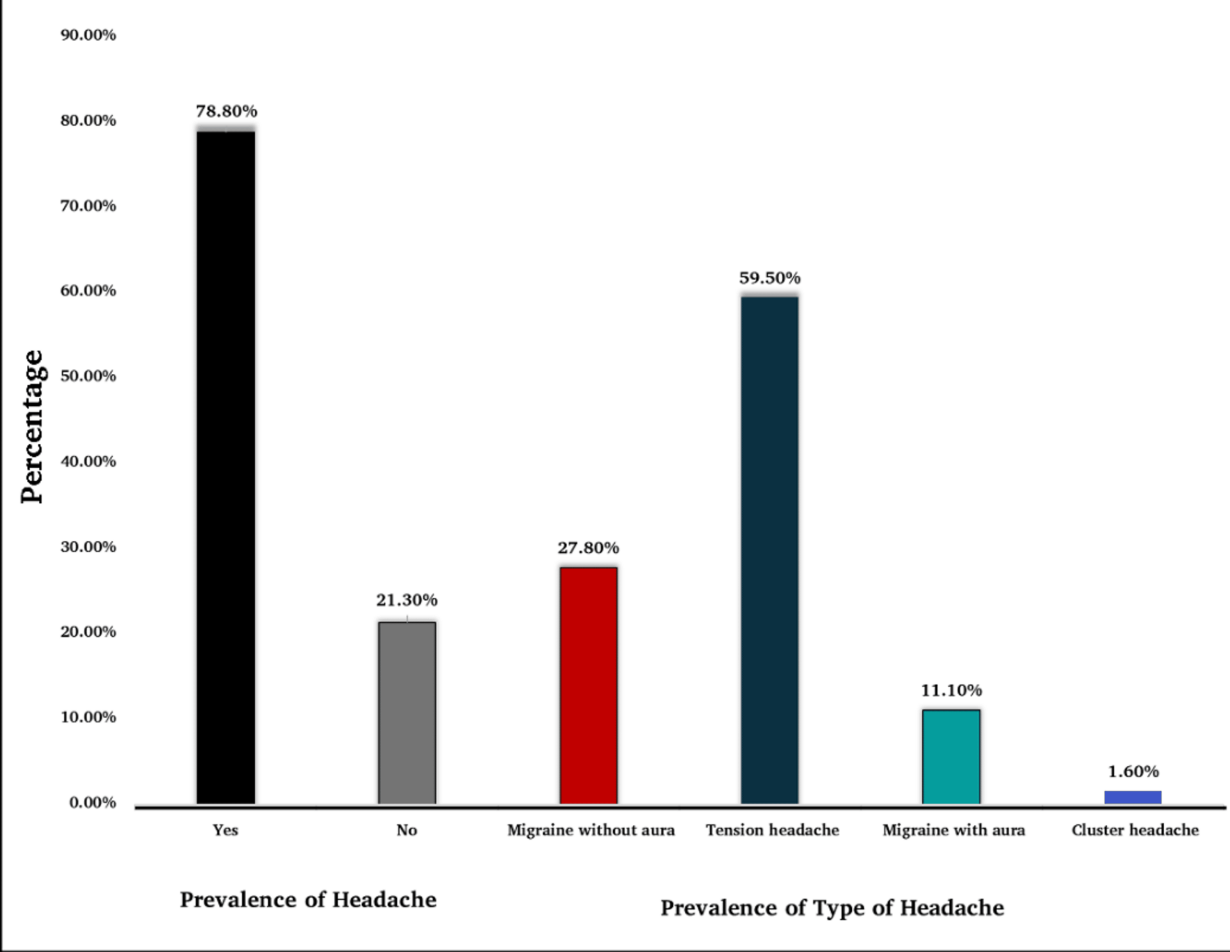
Prevalence of headaches and their subtype

The prevalence of headaches among medical students at our university was notably high, affecting approximately two-thirds of the participants (p-value = 0.000). Tension headache was the most prevalent type (Figure 1). Female participants exhibited a higher prevalence of headaches compared to males, with tension headaches being the most common type in both sexes. Among academic years, second-year students had the highest prevalence of headaches, with tension headaches being the most common across all academic years, except the 4th year, where migraine without aura was more prevalent (Table 2).

**Table 2:** Prevalence of headaches and their subtypes classified by sex and academic years (n=160) Note: The chi-square test is applied to show the association, and a p-value of <0.05 is considered significant.

Variables	Male n (%)	Female n (%)	1^st^ Year n (%)	2^nd^ Year n (%)	3^rd^ Year n (%)	4^th^ Year n (%)	5^th^ Year n (%)	6^th^ Year n (%)
Prevalence								
Yes	53 (42.1%)	73 (57.9%)	11 (8.7%)	41 (32.5%)	16 (12.7%)	17 (13.5%)	15 (11.9%)	26 (20.6%)
No	25 (73.5%)	9 (26.5%)	1 (2.9%)	14 (41.2%)	4 (11.8%)	4 (11.8%)	0 (0%)	11 (32.4%)
P-value (chi-square)	0.001 (10.611)		0.186 (7.494)					
Prevalence of type of headache								
Migraine without aura	12 (22.6%)	23 (31.5%)	3 (27.3%)	8 (19.5%)	4 (25.0%)	7 (41.2%)	6 (40.0%)	7 (26.9%)
Tension headache	36 (67.9%)	39 (53.4%)	7 (63.6%)	29 (70.7%)	11 (68.8%)	5 (29.4%)	7 (46.7%)	16 (61.5%)
Migraine with aura	4 (7.5%)	10 (13.7%)	1 (9.1%)	4 (9.8%)	1 (6.3%)	5 (29.4%)	1 (6.7%)	2 (7.7%)
Cluster Headache	1 (1.9%)	1 (1.4%)	0 (0.0%)	0 (0.0%)	0 (0.0%)	0 (0.0%)	1 (6.7%)	1 (3.8%)
P-value (chi-square)	0.384 (3.051)		0.271 (17.851)					

Association of headaches and their subtypes with academic and lifestyle factors

Headache prevalence was associated with participants who studied for 14-28 hours per week (49.2%) and those who occasionally engaged in late-night study sessions (59.5%). Additionally, headaches were linked to academic performance satisfaction (51.6%) and the use of electronic devices (98.4%). Furthermore, headache occurrence was associated with participants who did not follow a particular diet (58.7%) or primarily consumed home-cooked meals (54.8%), those who drank more than 2 L of water per day (43.7%) or more than two cups of coffee per day (34.1%), and those who slept six to eight hours per day (61.9%). Moreover, a similar pattern was observed for the subtypes of headaches (Table 3).

**Table 3:** Association of subtypes of headaches with academic and lifestyle factors Note: A chi-square test is applied to show the association, and a p-value of <0.05 is considered significant.

Variables	Migraine without Aura n (%)	Tension Headache n (%)	Migraine with Aura n (%)	Cluster Headache n (%)	P-value (chi-square)
Study Hours/Week					
<7 hours	4 (11.4%)	10 (13.3%)	0	6 (16.7%)	0.744 (5.962)
14-28 hours	19 (54.3%)	34 (45.3%)	8 (57.1%)	12 (33.3%)	
35-42 hours	10 (28.6%)	20 (26.7%)	3 (21.4%)	10 (27.8%)	
> 42 hours	2 (5.7%)	11 (14.7%)	3 (21.4%)	8 (22.2%)	
Late-Night Study Frequency					
Never	6 (17.1%)	18 (24.0%)	3 (21.4%)	0 (0.0%)	0.422 (6.010)
Sometimes	19 (54.3%)	47 (62.7%)	7 (50.0%)	2 (100.0%)	
Always	10 (28.6%)	10 (13.3%)	4 (28.6%)	0 (0.0%)	
Academic Performance Satisfaction					
Yes	15 (42.9%)	40 (53.3%)	9 (64.3%)	1 (50.0%)	0.559 (2.066)
No	20 (57.1%)	35 (46.7%)	5 (35.7%)	1 (50.0%)	
Usage of Internet Devices for Study					
Yes	34 (97.1%)	75 (100.0%)	13 (92.9%)	2 (100.0%)	0.224 (4.369)
No	1 (2.9%)	0 (0.0%)	1 (7.1%)	0 (0.0%)	
Eating and Drinking Habits					
I follow a vegan diet	0 (0.0%)	4 (5.3%)	0 (0.0%)	0 (0.0%)	0.258 (24.760)
I follow a keto diet	0 (0.0%)	1 (1.3%)	1 (7.1%)	0 (0.0%)	
I usually eat home-cooked meals	20 (57.1%)	40 (53.3%)	8 (57.1%)	1 (50.0%)	
I eat fast food >4 times per week	7 (20.0%)	18 (24.0%)	4 (28.6%)	2 (100.0%)	
I don't follow any particular diet	22 (62.9%)	42 (56.0%)	8 (57.1%)	2 (100.0%)	
I drink >2 cups of coffee per day	16 (45.7%)	24 (32.0%)	2 (14.3%)	1 (50.0%)	
I drink >2L of water per day	11 (31.4%)	37 (49.3%)	7 (50.0%)	0 (0.0%)	
Sleeping Habits					
I have problems falling asleep	10 (28.6%)	20 (26.7%)	8 (57.1%)	1 (50.0%)	0.247 (18.308)
I have problems staying asleep	6 (17.1%)	8 (10.7%)	3 (21.4%)	0 (0.0%)	
I sleep less than 6 hours per day	9 (25.7%)	15 (20.0%)	4 (28.6%)	1 (50.0%)	
I sleep 6-8 hours per day	22 (62.9%)	50 (66.7%)	6 (42.9%)	0 (0.0%)	
I sleep more than 8 hours	5 (14.3%)	12 (16.0%)	4 (28.6%)	1 (50.0%)	
Exercise Frequency					
1-3 hours per week	14 (40.0%)	38 (50.7%)	4 (28.6%)	1 (50.0%)	0.289 (14.191)
4-6 hours per week	7 (20.0%)	16 (21.3%)	4 (28.6%)	0 (0.0%)	
6-9 hours per week	7 (20.0%)	4 (5.3%)	2 (14.3%)	0 (0.0%)	
>10 hours per week	0 (0.0%)	3 (4.0%)	2 (14.3%)	0 (0.0%)	
Never	7 (20.0%)	14 (18.7%)	2 (14.3%)	1 (50.0%)	

## Discussion

In this study, we found that headaches were extremely common among medical students at Bahcesehir University, with a prevalence of 78.80%. This figure falls within the range reported in existing literature, corroborating findings from previous research, such as the 81.8% prevalence reported by Anaya et al. [7]. However, it is important to note that some studies report much lower prevalence rates. For instance, a study conducted in Nepal by Bhattarai et al. found a prevalence of only 21.54% [8]. This discrepancy may be attributed to Bhattarai et al. utilizing a combination of questionnaire responses and physical examinations to confirm diagnoses, whereas our study relied solely on self-administered questionnaires, which could influence the reported prevalence rates. Additionally, our findings revealed that headache episodes were more commonly reported among female students at our university, aligning with existing literature (p=0.001) [7-10].

Our study revealed a higher prevalence of tension headaches, accounting for 59.50% of the assessed students, surpassing the prevalence of migraines in both sexes. This finding aligns with existing literature [7-13]. According to the International Classification of Headache Disorders (ICHD) criteria, tension-type headaches are characterized by a pressing or tightening quality and mild-to-moderate intensity and are often bilateral, unlike migraines, which are typically characterized by unilateral throbbing pain and moderate-to-severe intensity and are often associated with nausea, vomiting, or photophobia [14]. The prevalence of migraine in our study was 38.9%, which is somewhat consistent with results from studies conducted on university students in India (30.0%) and Egypt (32.5%) [11,15]. Lower migraine prevalence was also reported in Nepal (21.54%) and Palestine (22.0%) [7,8].

The analysis revealed that the highest rate of headache disorders was observed among second-year students. However, it is essential to consider that this finding may be influenced by the larger sample size compared to other academic years. The correlation between academic year and headache prevalence was not statistically significant (p=0.186). Contrary to other studies that identified the highest prevalence of headaches in third-year students, as this is the transition year between the basic science and clinical phases [9], our study did not observe this trend, even though these studies have the same curriculum as ours.

The most common pain pattern in our study was pressure/tightness, followed by pulsating sensations. This aligns with the findings of the study conducted by Qazi et al. [15]. Additionally, the most common location of pain was bilateral, followed by forehead pain, which is similar to the results reported by Ojini et al. [10]. Among the participants who reported experiencing headache episodes, a significant proportion - specifically, 74 out of 124 individuals - noted a gradual onset of symptoms. In terms of frequency, 76 out of 124 individuals experienced headaches monthly, 10 participants reported daily headache episodes, and the remaining students reported weekly occurrences.

In our questionnaire, several common precipitating factors for headache episodes emerged prominently among the responses. Bright light, missing a meal, upcoming stressful exams, menstrual cycle fluctuations, and sleep disturbances were identified as some of the most prevalent triggers reported by the participants. These findings provide valuable insights into the multifactorial nature of headache disorders among students.

We conducted a thorough assessment of various lifestyle factors contributing to the demanding routines of medical students, including daily study hours, exercise frequency, dependence on electronic devices, late-night studying, and sleeping habits. Despite examining these aspects, none of these factors showed a statistically significant difference in relation to the headache subtype.

Notably, academic satisfaction was higher among students without headache episodes, indicating a potential link between well-being and headache frequency. Additionally, the use of electronic devices for studying purposes was significantly higher among students with headache episodes, particularly among those reporting tension headaches. In fact, a staggering 100% of students with tension headaches rely on electronic devices for studying. This highlights the importance of understanding the role of screen time in exacerbating headache symptoms among students.

Moreover, our findings indicate an association between sleep patterns and headache episodes. Students reporting more than eight hours of sleep were predominantly those without headache episodes, whereas those experiencing difficulties falling or staying asleep were more prevalent among students with headache episodes. A similar pattern of headache frequency was identified in a study conducted by Qazi et al., where 40% of students reported fewer hours of sleep as an aggravating factor for their headache episodes [16].

A concerning trend emerged where 86 students (69.4%) who reported experiencing headache episodes opted not to consult a physician but instead resorted to self-medication, with the most common medications being nonsteroidal anti-inflammatory drugs (NSAIDs) and acetaminophen. This finding aligns with studies conducted by Bhattarai et al. [8] in Nepal and Ojini et al. [10] in Nigeria, underscoring a potential gap in healthcare-seeking behavior among affected medical students. This raises important concerns about the appropriateness and safety of self-management strategies for headache symptoms.

Study limitations

Although our study provides valuable insights into the prevalence of headache disorders among university students, it is important to note that we did not assess the potential effect of age differences on headache prevalence. Understanding how age may influence headache occurrence could provide further depth to our findings and inform targeted interventions tailored to specific age groups within the university population. Additionally, our study did not explore the potential influence of socioeconomic status on headache prevalence. However, it is important to acknowledge that our university student population largely shares a similar socioeconomic background. While this homogeneity simplifies certain analyses, it also limits our ability to draw conclusions about the impact of socioeconomic factors on headache occurrence. Moreover, our university has a highly diverse student population with students from various nationalities, but nationality was not included in the demographic data collected. This limits our ability to assess the potential influence of cultural or nationality-related factors on headache prevalence. Future research involving diverse socioeconomic and cultural groups would provide a more comprehensive understanding of the complex interplay between these factors and headache disorders. Furthermore, the small sample size and the fact that the study was conducted at a single institution limit the generalizability of our findings to the broader population. To enhance statistical power and ensure more robust conclusions, multicentric studies with larger sample sizes are recommended in the future.

## Conclusions

In summary, our study found a high prevalence of headache disorders among medical students, with a rate of 78.80%. Tension headaches were more common than migraines, and several lifestyle factors, including sleep patterns, exercise frequency, and electronic device usage, were associated with headache prevalence. However, none of these factors were statistically significant. Academic stress, particularly during the second and sixth years, was also observed as a potential contributor, but it did not reach statistical significance. Despite relying on self-reported data and not assessing age and socioeconomic status, our findings highlight the need for targeted interventions to reduce stress and promote healthy habits among students. The high rate of self-medication underscores the necessity for improved healthcare-seeking behavior. Future research should incorporate physical examinations and explore the impact of age and socioeconomic factors to provide a more comprehensive understanding of headache disorders in this population.
